# Distinguishing Between Embryonic Provisioning Strategies in Teleost Fishes Using a Threshold Value for Parentotrophy

**DOI:** 10.3390/biom13010166

**Published:** 2023-01-13

**Authors:** Zoe M. G. Skalkos, James U. Van Dyke, Camilla M. Whittington

**Affiliations:** 1 School of Life and Environmental Sciences, The University of Sydney, Heydon-Laurence Building (A08), Sydney, NSW 2006, Australia; 2 School of Agriculture, Biomedicine and Environment, La Trobe University, Wodonga, VIC 3690, Australia

**Keywords:** matrotrophy, lecithotrophy, matrotrophy index, embryonic nutrition, viviparous, oviparous, patrotrophy, maternal–foetal interactions

## Abstract

The source of embryonic nutrition for development varies across teleost fishes. A parentotrophy index (ratio of neonate: ovulated egg dry mass) is often used to determine provisioning strategy, but the methodologies used vary across studies. The variation in source and preservation of tissue, staging of embryos, and estimation approach impedes our ability to discern between methodological and biological differences in parentotrophy indices inter- and intra-specifically. The threshold value used to distinguish between lecithotrophy and parentotrophy (0.6–1) differs considerably across studies. The lack of a standardised approach in definition and application of parentotrophy indices has contributed to inconsistent classifications of provisioning strategy. Consistency in both methodology used to obtain a parentotrophy index, and in the classification of provisioning strategy using a threshold value are essential to reliably distinguish between provisioning strategies in teleosts. We discuss alternative methods for determining parentotrophy and suggest consistent standards for obtaining and interpreting parentotrophy indices.

## 1. Introduction

Embryonic nutrition can be provided entirely from the maternally supplied yolk (lecithotrophy), or supplemented with post-fertilisation maternal (matrotrophy) [1,2], or paternal (patrotrophy) nutrient provisioning [1,3]. Lecithotrophy and parentotrophy represent endpoints of a complex continuum of embryonic nutritional patterns, with no definitive biological point of distinguishment [4,5], For example, small amounts of inorganic nutrient provisioning (calcium) occur in lecithotrophic-classified viviparous squamates, either mobilised from the eggshell, or via simple placentotrophy [6,7]. Squamate classification of provisioning strategy is often also associated with anatomy, i.e., simple vs. complex placentation for lecithotrophy and placentotrophy (parentotrophy via placenta), respectively, despite both capable of post-fertilisation nutrient provisioning [7]. The transport of inorganic molecules requires relatively simple mechanisms compared to the transport of organic molecules [8]. Interestingly though, small uptake of organic molecules (amino acids) from the external environment has also been observed in oviparous teleosts [4]. This suggests the ability of parentotrophic embryos to absorb nutrients from their environment may be retained from an egg-laying ancestor. This ability is a prerequisite for parentotrophy, when the absorbed nutrients are acquired from the gestating parent during pregnancy [1,2]. Applying two broad provisioning strategies to vertebrates that exhibit a continuum of nutrient provisioning abilities between lecithotrophy and parentotrophy, makes correctness and consistency difficult.

Here, we define parentotrophy as the paternal or maternal post-fertilisation supplementation of nutrients to developing embryos during gestation. The parentotrophy provisioning continuum amongst viviparous (live-bearing) teleosts ranges from incipient (mostly yolk reliant with small amounts of supplementation) to substantial parental supplementation post-fertilisation (primarily provisioning) [1,9], and can be achieved via many physiological processes [10]. Viviparous animals can exhibit any provisioning strategy along this continuum. In contrast, oviparous (egg-laying) embryos are lecithotrophic, except for monotremes, and some species that brood their embryos [1,9,11]. Brooding involves egg incubation on or in the parental body [11,12], and provides an opportunity for additional nutrients to be supplied by the brooding parent.

Teleosts (modern bony fishes) demonstrate a diverse range of parental care and reproductive strategies [13], including oviparity, viviparity (incubation inside the female reproductive tract [14], and oviparity with brooding in either parent. Thus, brooding males can provide embryonic nutrition in some species. For example, the Syngnathidae family (pipefish, seadragons, and seahorses) exhibits a specialised form of parental care, whereby fertilisation and gestation occur inside the male, in a specialised structure called the brood pouch [13,15]. Some syngnathids are capable of post-fertilisation provisioning from the father [3,16]. Thus, in this review we use the term parentotrophy to encompass both maternal and paternal post-fertilisation provisioning [10,11]. Unlike viviparous amphibians, amniotes, and chondrichthyans, viviparous teleosts do not develop Müllerian ducts during embryogenesis, thus there is no development of oviducts or uteri in females [10,17,18,19]. Consequently, viviparous teleosts have intra-ovarian rather than intra-uterine gestation [10]. 

Parentotrophy has independently evolved from the ancestral state, lecithotrophy [9], in at least 33 clades of vertebrates, with substantial parentotrophy evolving 24 times, mostly in bony fishes [1,20]. Matrotrophy has been most extensively studied in the viviparous teleost families; Anablepidae [21], Goodeidae [22] and Poeciliidae [23,24]. Anablepidae and Goodeidae exhibit extreme matrotrophy, increasing in dry mass one thousandfold over embryonic development [21,22]. In contrast, poeciliid embryos receive varying amounts of post-fertilisation nutrition ranging from a decrease in dry mass of 57% to an increase of 625% [9,25,26]. Patrotrophy has evolved at least once in teleosts in the Syngnathidae family [3], but has not yet been studied in other male-brooding teleosts, for example, the forehead-brooding Kurtidae [27], or mouth-brooding Apogonidae [27] and Ariidae [28]. 

The degree of parentotrophy is commonly measured using a matrotrophy index (MI) (or patrotrophy index (PI) in male pregnant species) [3,23,29]. This ratio has been applied to reptiles [30,31], sharks [32] and bony fishes [3,33]. Wourms et al. [2] were the first to suggest that the degree of post-fertilisation nutrient provisioning could be measured by “the ratio of dry weight of the developed embryo to that of the fertilised egg”. This notion has since evolved to the dry mass of the neonate or newborn at birth divided by the dry mass of the recently fertilised egg [24,34]. The ratio is referred to in this review as matrotrophy index (MI) in matrotrophic species and patrotrophy index (PI) in patrotrophic species. As weight is relative to gravity and the studies mentioned here measured mass, we use the term, mass, for accuracy and consistency.

The dry mass lost by embryos during development in oviparous species provides a threshold MI or PI value used to distinguish between lecithotrophic and parentotrophic nutrition. In oviparous teleosts, the catabolised portion of the yolk to provide energy for growth results in a dry mass loss; thus, the average ratio of an oviparous teleost is MI = 0.7, but always <1, as the embryonic nutrition is provided in the yolk or eggshell only [23,35]. A lecithotrophic viviparous MI is expected to be similar to oviparous teleosts, as neither receive post-fertilisation provisioning. 

Despite the continuum of embryonic provisioning, the degree of dry mass lost in oviparous species is used as a threshold value above which a closely-related viviparous species is assumed parentotrophic. However, the threshold values used to distinguish between lecithotrophy and parentotrophy vary considerably across the teleost literature (anything between 0.6 and 1.0: see Section 2), resulting in inconsistent application of threshold values to classify viviparous or brooding species as lecithotrophic or parentotrophic. The concerns about the use of a threshold MI/PI are: (1) it assumes a consistent dry mass loss across oviparous species, but available data are lacking for oviparous teleosts; (2) it assumes consistency in catabolic costs across lecithotrophic viviparous and oviparous species [36]; (3) parentotrophic species may catabolise nutrients at a higher rate if they are receiving additional parental supplementation [37]; and (4) there are varying methodologies for measuring MI/PI. A review of dry weight losses in chondrichthyans questions the current use of threshold MI value [38]. The authors found a large discrepancy in the standard chondrichthyan threshold MI = 0.8 and the MI 0.6 calculated for the oviparous *Heterodontus portusjacksoni* and discuss inconsistencies and inaccuracies in previous chondrichthyan research. Frazer et al. [38] strongly recommended the disuse of a threshold value to distinguish between lecithotrophy and incipient parentotrophy in chondrichthyans. Therefore, in this review, we evaluate the use of threshold values to classify parentotrophy in teleosts and discuss the variation in methodology used across studies. The goal of this review is to determine the applicability of a single threshold value indicating teleost parentotrophy, and to provide suggestions for methodological consistency to enable a meaningful threshold value to be determined.

## 2. Teleost Parentotrophy Indices: Methodology and Provisioning Strategy Classification

Teleost literature varies in its methodology of obtaining and interpreting a parentotrophy index for the purpose of classifying a provisioning strategy to a species (Table S1). It differs in the embryonic stages used, the source and preservation of samples, and the use of real or estimated means to calculate an MI/PI. This variation can result in parentotrophy indices not accurately representing real differences in dry mass between birth and fertilisation and eliminate the ability to compare results between studies. Furthermore, the interpretation of an MI/PI varies across studies using varying parentotrophy thresholds and statistical methods, resulting in an overlap of provisioning strategy classifications between and within species. Here, we explore sources of variation in calculating MI/PI.

### 2.1. Methodological Sources of Variation in Parentotrophy Indices

#### 2.1.1. Raw vs. Regression Estimated Means

An MI/PI can be calculated from either raw (real) or estimated data for the dry mass of an offspring at birth divided by the raw or estimated dry mass of an embryo at fertilisation. Thus, dry masses are required from two embryonic developmental stages: “near-fertilisation” and “near-birth”. Raw data are derived from measuring dry masses of either preserved or freshly collected samples. For these comparisons, mean dry masses from several to hundreds of embryos/offspring are calculated (see references in Table 1).

Studies using raw data to measure dry mass means do so through varying methods to pool offspring within a clutch. These methods include: whole brood total dry mass divided by the number of offspring per brood [39,40,41]; dividing the whole clutch into pools of two to ten individuals for dry mass measurement, dividing each result by the number of offspring in each pool, and then calculating a clutch average [3,42]; or measuring a predetermined number of pooled offspring per clutch (5-100) [33,43]. Some studies do not specify if or how they pooled, subsampled or used whole clutches to measure dry mass, and each study has varying numbers of replicates per stage of development.

In contrast, estimating mean dry mass data uses dry mass data from varying (often opportunistic) stages of embryonic development to code either a linear or quadratic regression model of the relationship between embryonic stage and dry mass. These models are used to estimate the dry masses of embryos at fertilisation and/or at birth and are suitable when embryos cannot be collected at those specific developmental stages. The predicted values, rather than raw measured values, are then used to calculate the parentotrophy index.

Using regression models to predict embryonic dry mass is flawed for two main reasons. Firstly, a regression assumes that the *x*-axis values are truly quantitative and continuous. Embryonic stage is a discrete variable and offers a qualitative descriptor of the progression of embryogenesis [44,45]. Using embryonic stage as the independent variable assumes a consistent quantitative progression between each stage from beginning to end, which is unlikely [44]. Time after fertilisation or prior to birth, rather than embryonic stage, could be a better consistent quantitative metric, but is still not ideal as developmental rate is affected by environmental variables like temperature [46,47]. Furthermore, a regression of mass vs. time would demonstrate embryo mass gain or loss over time, which does not equate to a parentotrophy index. 

The second problem with predicting *y*-axis values from a regression is that predictions are only valid for the range of data used to estimate the model. Thus, attempts to estimate fertilised egg or newborn dry mass from regressions based on datasets comprising developmental stages after fertilisation and prior to birth will accumulate error at the extremes of the developmental stages as predictions stray further from the available data on the *x*-axis. Regression models could be improved by including mature eggs prior to fertilization, and newborns. Yet, many teleost studies use a regression to estimate MI/PI but do not specify using samples that expands that full range (see references in Table 1). Furthermore, very few studies provide the sample sizes for each embryonic stage the regression was based on. For those that do, the sample sizes at either extreme of embryonic development are very low. For example, out of a sample size of ~48 clutches of reproductively active *Heterophallus milleri*, only two and three clutches were from the earliest and latest embryonic stages, respectively, with most data collected from mid-development [48]. The timing of parentotrophy during embryonic development is variable between species (see Section 2.2.3). Therefore, making predictions outside the existing data range, especially with small sample sizes or no samples at the two available ends of development, is discouraged. 

Differences in real mean and estimated mean may result in incorrect provisioning strategy classification for species that are lecithotrophic or incipiently parentotrophic. Regressions are therefore likely not suitable for estimating dry masses using an MI/PI for many teleost species. In contrast, this approach is less likely to have a strong influence on conclusions drawn for highly parentotrophic species than for incipiently parentotrophic or lecithotrophic species, since the difference in fertilised egg and newborn dry mass is so large, e.g., *Dermogenys sumatrana*, which has an MI of 198.5 [49]. The regression approach is therefore potentially useful for such species, even when recently fertilised eggs or newborns are unavailable.

#### 2.1.2. Embryonic Staging

The ideal MI calculation is estimated as the dry mass of the offspring at birth divided by the dry mass of the embryo at fertilisation [2,24]. As yolk formation is complete by the time fertilisation occurs, any additional nutrients present in the embryo must be due to post-fertilisation provisioning [2,50]. However, the embryonic stages used to estimate an MI/PI from raw data vary across teleost studies.

Obtaining samples exactly at fertilisation or birth is impossible in some viviparous teleosts without constant monitoring, thus the next best achievable stage is often used. However, these stages are not consistent across studies, which is problematic in two ways. Firstly, the post-fertilisation sampling delay can affect a calculated MI/PI as parentotrophy may have already started. For lecithotrophic species, the later the sampling occurs after fertilisation, the more likely the embryos have already lost dry mass and thus, the MI would be overestimated. For parentotrophic species, a longer time post-fertilisation before sampling may result in an underestimated MI/PI, as provisioning may have already begun. There is no consistent standard for “near fertilisation” sampling within or across teleost studies, and timing varies considerably across fresh data studies from minutes [50], to hours [3]. Secondly, the amount of time before or after birth that samples are collected can also affect a calculated MI, with no consistent standard for “near birth”. Normal tables of development vary across species and thus, without consistent staging of both near fertilisation and near birth within a species or the equivalent between species, comparisons cannot be drawn. Most studies followed Haynes [44] embryonic staging, comparing stage four (blastocyst) to stage eleven (mature) [51,52], or Reznick [45], comparing stage two (un-eyed) to stage six (very-late eyed/mature) [42,53]. Stage four and eleven from Haynes [44], equate to stage one and six in Reznick [45], respectively. Using late-stage embryos is common for wild populations as we predict collection of this stage prior to birth, reduces the potential effect of catabolism or consumption of food from the external environment, on new-born dry mass and is more logistically achievable. In contrast, laboratory studies can sample immediately after birth [54], or within hours of parturition [3]. The point at which parental nutrient transport to embryos starts and the constant or fluctuating rate at which it occurs is unknown for most parentotrophic teleosts, so the measurable impacts of sampling post-fertilisation or pre-birth are unknown. Even so, using near birth and near fertilisation stages likely results in under- or over-estimated MIs, and studies using different methodology in their embryonic staging are not comparable. 

#### 2.1.3. Variation in Maternal Size 

Ideally, a parentotrophy index comparison of embryos at fertilisation and new-borns would be obtained from the same female, for multiple individuals across multiple populations. This is possible for species with superfetation, where two or more broods are developing at the same time within the same female, but collecting multiple individuals containing both “near fertilisation” and “near-birth” embryonic stages is likely to be difficult. Thus, matrotrophy indices compare the eggs from multiple females to the offspring of multiple other females, assuming heterogeneity of initial egg size. However, larger egg [55,56] and new-born [56,57,58,59,60] dry mass/size is correlated with larger viviparous females in some species. Note that an increase in offspring size does not always equate to an increase in offspring dry mass [33]. Furthermore, the degree of parentotrophy may change with female size/age [61]. The influence of maternal size can be methodologically mitigated by selecting for same age/sized females across embryonic comparisons. 

#### 2.1.4. Source and Preservation of Samples

Dry mass measurements used to calculate both raw and estimated MI/PI can be derived from wild or captive, fresh or preserved samples, which can alter the dry mass of a sample. Only two studies within the viviparous teleost MI/PI range of 0.6 and 1.1 used fresh samples, both in laboratory conditions [3,54]. All oviparous teleost studies mentioned here measured dry mass changes from fresh samples, with liquid nitrogen used as temporary storage for *Danio rerio* [62], before drying (Table 1). However, no significant difference is observed between dry masses of demersal fish eggs frozen before drying and eggs dried fresh [63]. Most viviparous studies presented here use samples from wild populations with preserved specimens in Neutral Buffered Formalin (NBF) alone or followed by ethanol, in varying concentrations (Table 1). However, preservation in formaldehyde solutions can significantly increase [64] or decrease [63,65] fish egg dry mass. The use of NBF can also result in some loss of lipids from tissue [66]. Furthermore, as lipids are ethanol soluble [67], fixation in ethanol measures lean dry mass, which can be different to dry mass [68,69]. The use of these preservatives can result in a conservative or exaggerated parentotrophy index. Not all teleost parentotrophy research specifies or measures the effects of the method of preservation, except for Thibault and Shultz [43] (Table 1). For example, Olivera-Tlahuel et al. [70] obtained data from alternate sources to use in their MI regression formula when calculating MIs for some species. For two of those species, *Phalloceros caudiomaculatus* [68] and *Phalloceros anisophallos* [71], the data obtained were lean dry masses, but the MIs were presented with those derived from dry masses, without distinguishment. Studies in which samples were preserved in NBF or ethanol may not accurately represent the true MI/PI, unless validated by determining the effect of preservation on the embryo dry mass. This is important when distinguishing between facultative or incipient parentotrophy and lecithotrophy because any preservative effect on dry mass may result in the classification of an incorrect provisioning strategy.

**Table 1:** Parentotrophy indices calculated for teleost fishes indicate discrepant application of a threshold value for parentotrophy.

Species	MI/PI	Real v Estimated	Parentotrophy Threshold	Statistical Test for Significant Difference	Classification of Nutrient Provisioning Strategy	Resource
Viviparous						
*Alfaro huberi*	0.64	EEN	>1	NA	Lecithotrophy	[72] ^P^
*Belonesox belizanus*	0.70	EEN	>1	NA	Lecithotrophy	* [72] ^P^
*Brachyrhaphis episcopi*	0.78	R	Not stated	NA	Not specified	[73] ^PF^
*Brachyrhaphis holdridgei*	0.66	EEN	>1	NA	Lecithotrophy	[72] ^P^
*Brachyrhaphis rhabdophora*	0.77	EEN	>1	NA	Lecithotrophy	* [72] ^P^
*Dermogenys burmanica*	0.67	EEN	>0.7	0.7	Lecithotrophy	[49] ^PE^
*Dermogenys siamensis*	0.64	EEN	>0.7	0.7	Lecithotrophy	[49] ^PE^
*Gambusia affinis*	0.62	EEN	>1	NA	Lecithotrophy	* [72] ^P^
*Gambusia aurata*	0.82	EEN	≥0.8	NA	Matrotrophy	[70] ^PE^
*Gambusia holbrooki*	0.70	R	Not stated	NA	Not specified	** [42]
	0.64	EEN	>1	NA	Lecithotrophy	* [72] ^P^
*Gambusia hubbsi*	0.86	EN	>0.7	0.7	Both	** [74] ^PE^
*Gambusia punctata*	0.78	EEN	>0.7	0.7	Lecithotrophy	[75] ^P^
*Gambusia sexradiata*	0.73	EEN	>0.7	0.7	Lecithotrophy	[75] ^P^
*Gambusia vittata*	0.77	EEN	>0.7	0.7	Lecithotrophy	** [75] ^P^
	0.74	EEN	>1	NA	Lecithotrophy	[72] ^P^
	1.29	EEN	≥1	NA	Both	** [41] ^PE^
*Gambusia wrayi*	0.70	EEN	>0.7	0.7	Lecithotrophy	[75] ^P^
*Hemirhamphodon kapuasensis*	0.61	EEN	>0.7	0.7	Lecithotrophy	[49] ^PE^
*Hemirhamphodon pogonognathus*	0.64	EEN	>0.7	0.7	Lecithotrophy	[49] ^PE^
*Heterophallus milleri*	0.74	EEN	>0.75	NA	Lecithotrophy	[48] ^PF^
*Hippocampus abdominalis_p_*	1	R	>0.7	Stage	Patrotrophy	[3] ^F^
*Hippocampus fuscus_p_*	0.75	R	Not stated	NA	Not specified	[54] ^F^
*Limia dominicensis*	0.65	EEN	≥1	NA	Lecithotrophy	[76] ^P^
	0.51	EEN	≥1	NA	Lecithotrophy	[77] ^N^
*Limia heterandria*	0.67	EEN	≥1	NA	Lecithotrophy	[77] ^N^
*Limia melanogaster*	0.71	EEN	≥1	NA	Lecithotrophy	** [77] ^N^
	0.67	EEN	≥1	NA	Lecithotrophy	[76] ^P^
*Limia melanonotata*	0.67	EEN	≥1	NA	Lecithotrophy	[77] ^N^
*Limia nigrofasciata*	0.64	EEN	≥1	NA	Lecithotrophy	** [77] ^N^
*Limia pauciradiata*	0.66	EEN	≥1	NA	Lecithotrophy	[77] ^N^
*Limia perugiae*	0.90	EEN	≥1	NA	Lecithotrophy	[77] ^N^
*Limia tridens*	0.90	EEN	≥1	NA	Lecithotrophy	[77] ^N^
*Limia versicolor*	0.74	EEN	≥1	NA	Lecithotrophy	[77] ^N^
*Limia vittata*	0.76	EEN	≥1	NA	Lecithotrophy	** [77] ^N^
*Limia zonata*	0.91	EEN	≥1	NA	Lecithotrophy	[77] ^N^
*Micropoecilia picta*	0.78	EEN	>0.7	0.7	Lecithotrophy	** [78] ^PF^
*Nomorhamphus kolonodalensis*	0.66	EEN	>0.7	0.7	Lecithotrophy	[49] ^PE^
*Nomorhamphus megarrhamphus*	0.84	EEN	>0.7	0.7	Lecithotrophy	[49] ^PE^
*Nomorhamphus weberi*	0.77	EEN	>0.7	0.7	Lecithotrophy	[49] ^PE^
*Phallichthys fairweatheri*	0.65	EEN	≥0.7	0.7	Lecithotrophy	[25] ^P^
*Phallichthys quadripunctatus*	0.75	EEN	≥0.7	0.7	Lecithotrophy	** [25] ^N^
*Poecilia caucana*	0.77	EEN	≥1	NA	Lecithotrophy	[76] ^P^
*Poecilia latipinna*	0.92	EEN	Not stated	NA	Both	** [79] ^PF^
*Poecilia latipunctata*	0.85	EEN	≥1	NA	Lecithotrophy	[80] ^PF^
*Poecilia mexicana*	0.63	EN	>1	NA	Lecithotrophy	[72] ^P^
	0.57	EEN	>0.7	NA	Lecithotrophy	** [81] ^PF^
	0.68	EN	>0.65	NA	Lecithotrophy	** [34] ^PF^
*Poecilia reticulata*	0.70	EEN	>0.7	0.7	Lecithotrophy	** [78] ^PF^
*Poecilia wingei*	0.84	EEN	>0.7	0.7	Lecithotrophy	[78] ^PF^
*Poeciliopsis baenschi*	0.98	EEN	≥0.8	NA	Matrotrophy	[70] ^PE^
*Poeciliopsis balsas*	1.05	EN	>0.6	0.7	Lecithotrophy	[23] ^P^
*Poeciliopsis catemaco*	0.68	EN	>0.6	0.7	Lecithotrophy	[23] ^P^
*Poeciliopsis fasciata*	0.81	EN	>0.6	0.7	Lecithotrophy	[23] ^P^
*Poeciliopsis gracilis*	0.69	EN	>0.6	0.7	Lecithotrophy	[23] ^P^
	0.84	EEN	≥0.8	NA	Matrotrophy	[70] ^PE^
	0.80	EEN	≥1	NA	Both	** [82] ^PE^
	0.72	R	Not stated	NA	Lecithotrophy	[51] ^PE^
*Poeciliopsis hnilickai*	0.86	EN	>0.6	0.7	Lecithotrophy	[23] ^P^
*Poeciliopsis infans*	0.86	EN	>0.6	0.7	Lecithotrophy	[23] ^P^
	1.05	EEN	≥0.8	NA	Matrotrophy	[70] ^PE^
*Poeciliopsis latidens*	0.86	EN	>0.6	0.7	Matrotrophy	[23] ^P^
*Poeciliopsis monacha*	0.61	R	Not stated	NA	Not specified	[43] ^PE^
*Poeciliopsis scarlli*	0.87	EN	>0.6	0.7	Lecithotrophy	[23] ^P^
*Poeciliopsis turrubarensis*	0.66	EN	>0.6	0.7	Lecithotrophy	[23] ^P^
	1.05	R	Not stated	NA	Matrotrophy	** [52] ^PE^
*Poeciliopsis viriosa*	0.93	EN	>0.6	0.7	Matrotrophy	[23] ^P^
*Priapella chamulae*	0.71	EEN	>0.75	Stage	Lecithotrophy	[34] ^PF^
*Priapella intermedia*	1.03	R	Not stated	NA	Matrotrophy	[51] ^PE^
*Priapella olmecae*	0.76	EEN	≥0.8	NA	Lecithotrophy	[70]
*Priapichthys festae*	0.60	R	>0.65	NA	Lecithotrophy	[83] ^PF^
*Pseudoxhiphophorus jonesii*	0.65	EEN	≥0.8	NA	Lecithotrophy	[70] ^PE^
*Syngnathus schlegeli_p_*	0.71	R	Not stated	Stage	Patrotrophy	[33] ^PE^
*Xiphophorus hellerii*	0.61	EEN	>1	NA	Lecithotrophy	[72] ^P^
Oviparous						
*Clupea harengus*	0.73	R	Not stated	NA	Not specified	[84] ^F^
*Danio rerio*	0.77	R	Not stated	NA	Not specified	[62] ^FL^
*Salmo fario*	0.63	R	Not stated	NA	Not specified	[50] ^F^
*Salmo salar*	0.70	R	Not stated	NA	Not specified	[85] ^F^
*Salmo irideus*	0.62	R	Not stated	NA	Not specified	[86] ^F^
*Salvelinus fontinalis*	0.75	R	Not stated	NA	Not specified	[87] ^F^

Only MI/PI between 0.6 and 1.1 were included to represent the species most likely affected by in-consistencies in methodology. The calculated matrotrophy index (MI) or patrotrophy index (PI) and the classification of nutrient provisioning strategy is shown for each study. Each of the included studies calculates at least one Parentotrophy Index (MI/PI) or % dry mass change during embryogenesis. For studies containing multiple populations, the average is displayed here (**). Estimations were performed using regression models. Statistics listed were parentotrophy index-specific in determining provisioning strategy. Parentotrophy index values were rounded to 2 decimal places. * = not original data source, but MI is reported in the publication. In the Test for Significant Difference column, ”stage” indicates a test for significant difference between early and late/newborn stage and “0.7” indicates testing the MI/PI to be significantly different to 0.7. (NA) = no classifying statistics performed or no parentotrophy threshold given and thus no classification; (R) = raw mean embryo dry mass values from two embryonic stages (fresh and pre-served with 10% Neutral Buffered Formalin) were used to calculate the MI/PI; (EN) = regression used to estimate newborn dry mass; EEN = regression used to estimate embryo at fertilisation and newborn dry mass. Subscript (p) indicates a Patrotrophy Index for male gestating parents. Superscripts indicate whether samples were fresh (F), fresh but briefly stored in liquid nitrogen (FL), preserved in Neutral Buffered Formalin (PF), preserved in Ethanol with or without formalin fixation (PE), preserved but methodology not specified (P), or not specified whether fresh or preserved (N). All preservation types were included for oviparous due to the limited studies available. Ash-free (organic) masses were not included. Studies not specifying fresh or pre-served samples were not included. Studies that used unfertilised eggs as comparison were excluded unless they specified that they were mature. Thresholds and classifications were only included if specified in the original source.

### 2.2. Methodological Sources of Variation in Provisioning Strategy Classification

#### 2.2.1. Discrepant Use of Threshold Value for Parentotrophy

**Figure 1:**
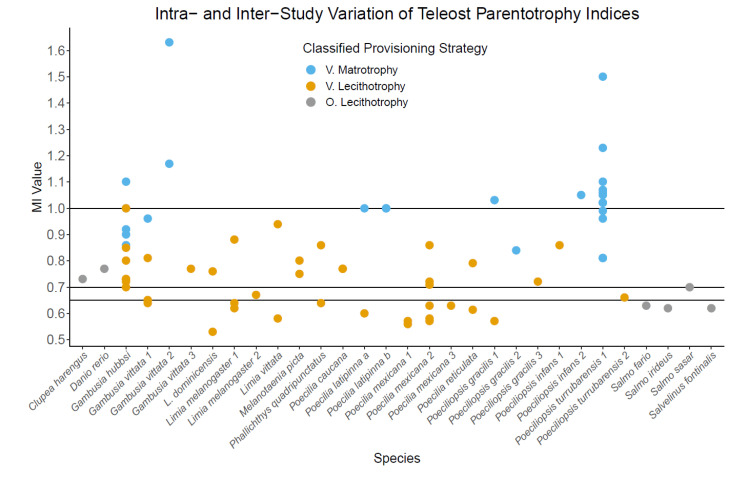
The variation in matrotrophy indices and parentotrophy classifications between populations of teleosts. Each species along the x-axis represents a single study, except for *Poecilia latipinna* (a & b), which come from a single study and represent two populations (population a & population b) one year apart. If there are multiple studies for a single species, each study is separated by species name followed by a number (1–3). Each plotted value for each species represents a separate population within a study, except for *Poeciliopsis gracilis* 1, which represents the same population across the wet season (classified as lecithotrophy) and dry season (classified as matrotrophy), and *Poecilia latipinna*. All viviparous MI values were estimated using regression models except for *Poeciliopsis turrubararensis* and all oviparous species, which used raw mean values. *Poecilia caucana* includes two populations with an MI of 0.77. The provisioning strategy presented was assigned by each paper based on their specified threshold and statistics. The varying threshold MI and PI values used to classify parentotrophy in different studies are >0.65, >0.7, or ≥1, represented by horizontal lines. There was either no statistics or confirmation of parentotrophy was done by determining whether the population MI was significantly different to 0.7 (refer to Table 1). (V.) represents viviparous species in which an MI has been calculated for at least two populations. (O.) represents oviparous, lecithotrophic species, which were included for comparison.

The threshold value for parentotrophy used in teleost literature is highly variable, ranging from MI > 0.6 to MI > 1 (Table 1), resulting in overlap of classifications along the provisioning continuum (Figure 1). The most common MI/PI threshold values are ≥1 and >0.7 (Table 1). The lack of a consistent threshold applied to the continuum of embryonic provisioning, means that distinguishing lecithotrophy from incipient parentotrophy is difficult and has resulted in overlap of classifications (Figure 1). Multiple species are classified differently across studies due to varying threshold values, including *Gambusia vittata*, *Poeciliopsis gracilis*, *Poeciliopsis infans*, and *Poeciliopsis turrubarensis* (Table 1, Figure 1). For teleosts with moderate or substantial parentotrophy (Table 2), the use of a consistent methodology is not as essential because the methodological causes of variation should not change the provisioning strategy classification. 

#### 2.2.2. Variation in Use of Statistical Tests

The lack of a standard method or statistical approach to assign a provisioning strategy based on an MI/PI can also account for some of the inconsistency in classification observed inter- and intraspecifically (Figure 1; Table 1). Most studies perform no parentotrophy index-specific statistics while others tested if the MI/PI was significantly different from 0.7. This can result in species with MI/PI values larger than 0.7 being classified as lecithotrophic, e.g., *Gambusia punctata and Micropoecilia picta* (MI = 0.78), or *Nomorhamphus megarrhamphus* (MI = 0.84) (Table 1), likely due to large variance around the mean and/or statistically inadequate sample sizes. The remaining studies assessed if their near fertilisation and near birth developmental stages were significantly different from one another (Table 1). 

#### 2.2.3. Intra-Specific Variation in Parentotrophy Indices

##### Intra-Specific Variation in MI/PI Suggests Parentotrophy

In lecithotrophic species, a change in biological factors (e.g., temperature, resource availability) should result in no change in calculated MI/PI across study conditions of populations, providing methodology is consistent and sample size is sufficient. However, if the species is capable of parentotrophy, then variation in MI/PI is likely to occur, even with consistent methodology, due to environmental or genetic factors. This variation means that under certain contexts, an MI could fail to detect parentotrophy, despite the species being parentotrophic. For example, if a species is facultatively parentotrophic, it would be unlikely to be detected in systems where the gestating parent does not have the excess nutrients/energy to provide to developing embryos, like in resource limiting environments [94,95]. As also seen in reptiles, if a pregnant mother has sufficient resource availability for facultative parentotrophy, the MI should increase [96]. For some teleost species with MIs calculated for multiple populations within a study, some populations are classified as lecithotrophic and others parentotrophic, for example *Gambusia hubbsi*, *G. vittata*, *P. gracilis*, and *Poecilia latipinna* (Figure 1). However, if a species is capable of parentotrophy, but does not always exhibit parentotrophy, then it should be classified as parentotrophic (i.e., capable of parentotrophy). By this standard, assuming methodology is consistent within studies, *G. hubbsi*, *G. vittata*, *P. gracilis*, and *P. latipinna* should all be classified as potentially parentotrophic. For species with MI/PI between 0.6 and 1, if methodology is not consistent within or across studies, large variation in parentotrophy indices may be methodological and incorrectly imply parentotrophy. We suggest that a parentotrophy index from a single population may not accurately represent a species’ ability for parentotrophy if its MI/PI falls within the range of 0.6 and 1, and that studies should look at multiple populations, age groups (or equal variation in parental ages across the two stages), and/or study conditions (e.g., resource availability).

##### Temporal Intra-Specific Variation in Parentotrophy

In parentotrophic teleosts, the timing, source, and quantity of embryonic nutrition across development is variable between species and can be non-linear. Species can receive parental provisioning at different stages of development, so the timing of sampling in species with varying metabolisms can heavily influence parentotrophy index results and provisioning conclusions. For example, Gambusia holbrooki is classified as lecithotrophic (Table 1), yet embryos increase in dry mass by almost 50% during embryogenesis before decreasing again before parturition [37]. This increase to ~150% of the original dry mass is significantly different to early and late embryonic masses. In contrast, in some Phalloceros species, the embryos decrease in dry mass at the beginning of gestation and then increase up to three-fold [90]. Similarly, Xenodexia ctenolepis embryos dry weights remain stable or decrease slightly until about half-way through gestation, where significant matrotrophy occurs, resulting in a three-to-four-fold increase in dry mass from fertilisation to birth [49]. Comparing two stages on either end of embryonic development without consideration for the intermediate stages, is a limitation to using the MI/PI to classify provisioning strategy and can result in incorrectly classifying a species as lecithotrophic. 

## 3. Alternative Approaches to Measuring Parentotrophy 

There are several alternative methods to determine provisioning strategies that have been used across vertebrate literature, including comparing the nutrient content of neonates to that of eggs via nutrient extractions, mass spectrometry or chemical composition analysis. In lecithotrophic species, if the nutrient in question is catabolisable (e.g., lipids), its mass should decrease due to the catabolism of the embryo. Thus, if transport of a metabolised nutrient occurs, then the mass of that nutrient in the newborns should be more than or slightly less than that of the recently fertilised eggs [3]. For example, quantifying lipids [3,97,98] and proteins [97,98] has been used as evidence for matrotrophy. 

To determine whether parentotrophy occurs, the transport of specifically labelled nutrients can be tracked between the parent and offspring, as commonly used in reptiles [98,99,100] and fish [16,94,101,102,103]. Currently, there are two direct labelling methods to track nutrient transport and determine the presence of parentotrophy: stable isotope labelled nutrients and radioactively labelled nutrients. These methods can be used with a range of nutrients including fatty acids [104] and amino acids [81,100,104] and consist of either feeding or directly injecting the labelled nutrients into pregnant individuals. The abundance of the labelled nutrients is then measured in the embryos to determine if they have taken up any of the labelled nutrients [104]. Labelled nutrient studies can offer opposite conclusions to a derived parentotrophy index. For example, an estimated MI of 0.56 and 0.57 for two separate populations of *Poecilia mexicana* suggests lecithotrophy [81]. However, the study also conducted a radio-tracer assay and found significant maternal nutrient transfer of labelled leucine to developing embryos, revealing parentotrophy had occurred. Respirometry by measurement of oxygen consumption, and calorimetry by measurement of heat loss, can indirectly measure embryonic metabolic rates and has been used to make inferences on the level of parentotrophy in the genus *Sebastes* [36]. Respirometry indicates that *Sebastes melanops* embryos catabolise 64% of the energy provided in the egg but offspring at birth contained 81% of the initial energy of the mature egg. Therefore, the total energy required for embryonic development is ~1.45 x the initial egg supply, suggesting that the species is parentotrophic [36]. Additionally, immunohistochemistry can localise proteins [105] involved in nutrient transport and can display how, when and/or where parentotrophy may be occurring.

## 4. Conclusions and Future Directions

Teleost nutrient provisioning strategies range from lecithotrophy to extreme parentotrophy [21,22], and distinguishing between the two is vital in gaining foundational biological understanding of a species’ reproduction. Therefore, methodological consistency in obtaining an MI/PI and consistency in a parentotrophy threshold is essential for establishing a credible standard parentotrophy index comparison across species, populations, and studies. We argue that preserved specimens may not accurately estimate an MI/PI unless validated with a comparison of dried fresh and dried preserved specimens. We further argue that calculating an MI/PI using a regression to estimate dry mass based on embryonic staging can be problematic because embryonic stage is not quantitative and continuous, and predictions cannot be made outside the range of data used to make the regression. We therefore propose that a single threshold cannot be used to distinguish between lecithotrophy and incipient parentotrophy in every teleost species, similar to Frazer et al.’s [38] recommendations for chondrichthyans. For species with 0.6 ≤ MI ≤ 1, we recommend the addition of another method to distinguish between lecithotrophy and parentotrophy (e.g., nutrient extractions or mass spectrometry of embryos/uterine or pouch fluid, radio-tracer assay, histology, and electron microscopy), to confirm a species’ ability (whether realised in natural populations or not) to be parentotrophic. Alternatively, studies of similar sized/aged animals in multiple environmental treatments (e.g., feeding regimes) in which separate parentotrophy indices are calculated may be useful to determine any variability that may suggest parentotrophy. The number of oviparous teleost species for which MI has been calculated is limited and these studies are highly variable in their methodology. Thus, further research is required with consistent embryonic stage comparisons between species and a preference for the use of fresh rather than fixed tissues. With consistent methodology, the biological and genetic causes for variation in parentotrophic species can then be compared across studies, species, and populations. 

## Figures and Tables

**Table 2:** A non-exhaustive list of published parentotrophy indices for teleost species with MI < 0.6 or MI > 1.1, including examples from all known matrotrophic teleost families in which MI/PI studies have been conducted; Anablepidae; Clinidae; Goodeidae; Poeciliidae; Zenarchopteridae. All data are presented as in the resource, to 2 decimal places.

Species	Matrotrophy Index	Classification of Nutrient Provisioning Strategy	Resource
*Ameca splendens*	150.00	Matrotrophy	[2]
*Clinus superciliosus*	35.60	Matrotrophy	[88]
*Dermogenys bispina*	152.00	Matrotrophy	[49]
*Dermogenys orientalis*	18.35	Matrotrophy	[49]
*Dermogenys sumatrana*	198.50	Matrotrophy	[49]
*Gambusia rhizophorae*	1.24	Matrotrophy	[75]
*Gambusia speciosa*	0.45	Lecithotrophy	[70]
*Gambusia yucatana*	0.53	Lecithotrophy	[70]
*Hemirhamphodon keukenthali*	0.58	Lecithotrophy	[49]
*Heterandria formosa*	14.18	Matrotrophy	[40]
*Jenysia multidentata*	606.14	Matrotrophy	[89]
*Limia caymanensis*	0.57	Lecithotrophy	[77]
*Micropoecilia bifurca*	55.06	Matrotrophy	[78]
*Micropoecilia branneri*	86.84	Matrotrophy	[78]
*Micropoecilia parae*	6.33	Matrotrophy	[78]
*Nomorhamphus bakeri*	3.44	Matrotrophy	[49]
*Nomorhamphus brembachi*	11.40	Matrotrophy	[49]
*Nomorhamphus manifesta*	15.80	Matrotrophy	[49]
*Nomorhamphus rossi*	22.00	Matrotrophy	[49]
*Phallichthys amates*	0.52	Lecithotrophy	[25]
*Phallichthys tico*	0.43	Lecithotrophy	[25]
*Phalloceros anisophallos*	2.80	Matrotrophy	[71]
	2.82	Matrotrophy	[90]
*Phalloceros aspilos*	2.50	Matrotrophy	[90]
*Phalloceros enneaktinos*	2.43	Matrotrophy	[90]
*Phalloceros harpagos*	3.33	Matrotrophy	[53]
	2.52	Matrotrophy	[90]
*Phalloceros leptokeras*	1.52	Matrotrophy	[90]
*Phalloceros tupinamba*	2.17	Matrotrophy	[90]
*Phalloceros wai*	2.64	Matrotrophy	[90]
*Pamphorichthys araguaiensis*	9.62	Matrotrophy	[76]
*Pamphorichthys hasemani*	36.37	Matrotrophy	[76]
*Pamphorichthys hollandi*	21.29	Matrotrophy	[76]
*Pamphorichthys minor*	1.63	Matrotrophy	[76]
*Pamphorichthys scalpridens*	16.58	Matrotrophy	[76]
*Poecilia butleri*	2.30	Matrotrophy	[91]
*Poeciliopsis elongata*	68.90	Matrotrophy	[23]
*Poeciliopsis lucida*	1.34	Matrotrophy	[43]
	1.79	Matrotrophy	[92]
*Poeciliopsis occidentalis*	1.12	Matrotrophy	[23]
	1.50	Matrotrophy	[39]
*Poeciliopsis pleurospilus*	0.50	Lecithotrophy	[70]
*Poeciliopsis presidionis*	21.5	Matrotrophy	[23]
*Poeciliopsis prolifica*	5.40	Matrotrophy	[23]
	7.01	Matrotrophy	[80]
*Poeciliopsis retropinna*	22.39	Matrotrophy	[93]
*Poeciliopsis turneri*	41.4	Matrotrophy	[23]
*Xenodexia ctenolepis*	3.86	Matrotrophy	[49]

## Data Availability

Not applicable.

## Supplementary Materials

The following supporting information can be downloaded at: https://www.mdpi.com/article/10.3390/biom13010166/s1, Table S1: Inclusion Criteria.
