# Supplementary material for: Distinguishing Between Embryonic Provisioning Strategies in Teleost Fishes Using a Threshold Value for Parentotrophy
Source: Biomolecules. 2023 Jan 13;13(1):166. doi: 10.3390/biom13010166 (PMC9856118; doi:10.3390/biom13010166)
Supplement: Supplementary file 1 [file biomolecules-13-00166-s001.zip › biomolecules-2041573-supplementary.pdf]

SUPPLEMENTARY MATERIAL

Table S1 Inclusion Criteria

Species	MI/PI	Real v Estimated	Parentotrophy Threshold	Statistical Test for Significant Difference	Classification of Nutrient Provisioning Strategy	Resource
Viviparous						
Alfaro huberi	0.64	EEN	>1	NA	Lecithotrophy	Pollux et al., 2014[P]
Belonesox belizanus	0.70	EEN	>1	NA	Lecithotrophy	*Pollux et al., 2014[P]
Brachyrhaphis episcopi	0.78	R	Not stated	NA	Not specified	Jennions and Telford, 2002[PF]
Brachyrhaphis holdridgei	0.66	EEN	>1	NA	Lecithotrophy	Pollux et al., 2014[P]
Brachyrhaphis rhabdophora	0.77	EEN	>1	NA	Lecithotrophy	*Pollux et al., 2014[P]
Dermogenys burmanica	0.67	EEN	>0.7	0.7	Lecithotrophy	Reznick et al., 2007[PE]
Dermogenys siamensis	0.64	EEN	>0.7	0.7	Lecithotrophy	Reznick et al., 2007[PE]
Gambusia affinis	0.62	EEN	>1	NA	Lecithotrophy	*Pollux et al., 2014[P]
Gambusia aurata	0.82	EEN	≥0.8	NA	Matrotrophy	Olivera-Tlahuel et al., 2015[PE]
Gambusia holbrooki	0.70	R	Not stated	NA	Not specified	**Fernández-Delgado & Rossomanno, 1997
	0.64	EEN	>1	NA	Lecithotrophy	*Pollux et al., 2014[P]
Gambusia hubbsi	0.86	EN	>0.7	0.7	Both	**Riesch et al., 2013[PE]
Gambusia punctata	0.78	EEN	>0.7	0.7	Lecithotrophy	Torres-Mejia, 2011[P]
Gambusia sexradiata	0.73	EEN	>0.7	0.7	Lecithotrophy	Torres-Mejia, 2011[P]
Gambusia vittata	0.77	EEN	>0.7	0.7	Lecithotrophy	**Torres-Mejia, 2011[P]
	0.74	EEN	>1	NA	Lecithotrophy	Pollux et al., 2014[P]
	1.29	EEN	≥1	NA	Both	**Weldele et al., 2014[PE]
Gambusia wrayi	0.70	EEN	>0.7	0.7	Lecithotrophy	Torres-Mejia, 2011[P]
Hemirhamphodon kapuasensis	0.61	EEN	>0.7	0.7	Lecithotrophy	Reznick et al., 2007[PE]
Hemirhamphodon pogonognathus	0.64	EEN	>0.7	0.7	Lecithotrophy	Reznick et al., 2007[PE]
Heterophallus milleri	0.74	EEN	>0.75	NA	Lecithotrophy	Riesch et al., 2011[PF]
Hippocampus abdominalis$_p$	1	R	>0.7	Stage	Patrotrophy	Skalkos et al., 2020[F]
Hippocampus fuscus$_p$	0.72	R	Not stated	NA	Not specified	Vincent, 1990[F]
Limia dominicensis	0.65	EEN	≥1	NA	Lecithotrophy	Pires & Reznick, 2018[P]
	0.51	EEN	≥1	NA	Lecithotrophy	Cohen et al., 2015[N]
Limia heterandria	0.67	EEN	≥1	NA	Lecithotrophy	Cohen et al., 2015[N]
Limia melanogaster	0.71	EEN	≥1	NA	Lecithotrophy	**Cohen et al., 2015[N]

	0.67	EEN	≥1	NA	Lecithotrophy	Pires & Reznick, 2018[P]
Limia melanonotata	0.67	EEN	≥1	NA	Lecithotrophy	Cohen et al., 2015[N]
Limia nigrofasciata	0.64	EEN	≥1	NA	Lecithotrophy	**Cohen et al., 2015[N]
Limia pauciradiata	0.66	EEN	≥1	NA	Lecithotrophy	Cohen et al., 2015[N]
Limia perugiae	0.90	EEN	≥1	NA	Lecithotrophy	Cohen et al., 2015[N]
Limia tridens	0.90	EEN	≥1	NA	Lecithotrophy	Cohen et al., 2015[N]
Limia versicolor	0.74	EEN	≥1	NA	Lecithotrophy	Cohen et al., 2015[N]
Limia vittata	0.76	EEN	≥1	NA	Lecithotrophy	**Cohen et al., 2015[N]
Limia zonata	0.91	EEN	≥1	NA	Lecithotrophy	Cohen et al., 2015[N]
Micropoecilia picta	0.78	EEN	>0.7	0.7	Lecithotrophy	**Pires et al., 2010[PF]
Nomorhamphus kolon-odalensis	0.66	EEN	>0.7	0.7	Lecithotrophy	Reznick et al., 2007[PE]
Nomorhamphus meg-arrhamphus	0.84	EEN	>0.7	0.7	Lecithotrophy	Reznick et al., 2007[PE]
Nomorhamphus weberi	0.77	EEN	>0.7	0.7	Lecithotrophy	Reznick et al., 2007[PE]
Phallichthys fairweath-eri	0.65	EEN	≥0.7	0.7	Lecithotrophy	Regus et al., 2013[P]
Phallichthys quad-ripunctatus	0.75	EEN	≥0.7	0.7	Lecithotrophy	**Regus et al., 2013[N]
Poecilia caucana	0.77	EEN	≥1	NA	Lecithotrophy	Pires & Reznick, 2018[P]
Poecilia latipinna	0.92	EEN	Not stated	NA	Both	**Trexler, 1985[PF]
Poecilia latipunctata	0.85	EEN	≥1	NA	Lecithotrophy	Pires, 2007[PF]
Poecilia mexicana	0.63	EN	>1	NA	Lecithotrophy	Pollux et al., 2014[P]
	0.57	EEN	>0.7	NA	Lecithotrophy	**Riesch, Plath, Schlupp & Marsh-Mathews, 2010[PF]
	0.68	EN	>0.65	NA	Lecithotrophy	**Riesch, Plath & Schlupp, 2010[PF]
Poecilia reticulata	0.70	EEN	>0.7	0.7	Lecithotrophy	**Pires et al., 2010[PF]
Poecilia wingei	0.84	EEN	>0.7	0.7	Lecithotrophy	Pires et al., 2010[PF]
Poeciliopsis baenschi	0.98	EEN	≥0.8	NA	Matrotrophy	Olivera-Tlahuel et al., 2015[PE]
Poeciliopsis balsas	1.05	EN	>0.6	0.7	Lecithotrophy	Reznick et al., 2002[P]
Poeciliopsis catemaco	0.68	EN	>0.6	0.7	Lecithotrophy	Reznick et al., 2002[P]
Poeciliopsis fasciata	0.81	EN	>0.6	0.7	Lecithotrophy	Reznick et al., 2002[P]
Poeciliopsis gracilis	0.69	EN	>0.6	0.7	Lecithotrophy	Reznick et al., 2002[P]
	0.84	EEN	≥0.8	NA	Matrotrophy	Olivera-Tlahuel et al., 2015[PE]
	0.80	EEN	≥1	NA	Both	**Molina-Moctezuma et al., 2019[PE]
	0.72	R	Not stated	NA	Lecithotrophy	Saleh-Subaie et al., 2021[PE]
Poeciliopsis hnilickai	0.86	EN	>0.6	0.7	Lecithotrophy	Reznick et al., 2002[P]
Poeciliopsis infans	0.86	EN	>0.6	0.7	Lecithotrophy	Reznick et al., 2002[P]
	1.05	EEN	≥0.8	NA	Matrotrophy	Olivera-Tlahuel et al., 2015[PE]
Poeciliopsis latidens	0.86	EN	>0.6	0.7	Matrotrophy	Reznick et al., 2002[P]

Poeciliopsis monacha	0.61	R	Not stated	NA	Not specified	Thibault & Schultz, 1978[PE]
Poeciliopsis scarlli	0.87	EN	>0.6	0.7	Lecithotrophy	Reznick et al., 2002[P]
Poeciliopsis turrubarensis	0.66	EN	>0.6	0.7	Lecithotrophy	Reznick et al., 2002[P]
	1.05	R	Not stated	NA	Matrotrophy	**Zúñiga-Vega et al., 2007[PE]
Poeciliopsis viriosa	0.93	EN	>0.6	0.7	Matrotrophy	Reznick et al., 2002[P]
Priapella chamulae	0.71	EEN	>0.75	Stage	Lecithotrophy	Riesch et al., 2012[PF]
Priapella intermedia	1.03	R	Not stated	NA	Matrotrophy	Saleh-Subaie et al., 2021[PE]
Priapella olmecae	0.76	EEN	≥0.8	NA	Lecithotrophy	Olivera-Tlahuel et al., 2015
Priapichthys festae	0.60	R	>0.65	NA	Lecithotrophy	Reznick et al., 1996[PF]
Pseudoxhiphophorus jonesii	0.65	EEN	≥0.8	NA	Lecithotrophy	Olivera-Tlahuel et al., 2015[PE]
Syngnathus schlegeli$_p$	0.71	R	Not stated	Stage	Patrotrophy	Watanabe &Watanabe, 2002[PE]
Xiphophorus hellerii	0.61	EEN	>1	NA	Lecithotrophy	Pollux et al., 2014[P]
Oviparous						
Clupea harengus	0.73	R	Not stated	NA	Not specified	Paffenhöfer & Rosenthall, 1968[F]
Danio rerio	0.77	R	Not stated	NA	Not specified	Hachicho et al., 2015[FL]
Salmo fario	0.63	R	Not stated	NA	Not specified	Gray, 1926[F]
Salmo salar	0.70	R	Not stated	NA	Not specified	Hayes & Armstrong, 1942[F]
Salmo irideus	0.62	R	Not stated	NA	Not specified	Smith, 1947[F]
Salvelinus fontinalis	0.75	R	Not stated	NA	Not specified	Gortner, 1913[F]

For viviparous species, only dry mass or lean mass was included, with wet mass and ash-free dry mass measurements excluded. Parentotrophy indices were not presented if stages of embryonic comparison were not consistent within a study (e.g., Pires et al. 2010 real data). Studies looking at hybridisation across species were excluded. Studies were excluded if they only graphically displayed dry mass data. For studies where raw data were not available, MI/PI calculations were not checked. Not all papers provided an MI/PI but provided dry mass data that could be used to calculate one. Both freeze-dried and oven-dried measures were included. Unfertilised egg comparisons were not used unless specified as mature or ovulated eggs. Due to the limited reference material of oviparous loss in dry mass, all available % loss in dry or lean dry mass in oviparous species was included. All calculations were performed with the number of decimal places provided in the data but were always rounded to 2 decimal places for the final MI value presented here. Calculations were checked if possible, and corrected if necessary. All provisioning classifications presented are as stated or suggested by the assigned threshold values in the original reference. We grouped all types of parentotrophy including incipient, moderate, substantial and their synonyms, into "parentotrophy". All species that presented an MI or PI between and including 0.6 and 1.1, were tabulated here, including species name(s), MI value, estimated or real MI/PI calculation, the threshold value in which parentotrophy is classified, any MI/PI specific statistical analysis, the classification given by the study and the source. We chose this range because most lecithotrophic or incipiently parentotrophic species fall within this range and it allows us to focus on the differences in distinguishing between the two across the literature.
